# Coordinating and Assisting Research at the SARS-CoV-2/Microbiome Nexus

**DOI:** 10.1128/mSystems.00999-20

**Published:** 2020-12-22

**Affiliations:** Colorado State University; Rutgers University; University of California, San Diego; University of California, San Diego; University of Colorado, Anschutz; University of Connecticut; University of California, Irvine; Colorado State University; University of Pittsburgh; DOE Joint Genome Institute; Johns Hopkins University; Duke University; Duke University; University of California, Irvine; Rutgers University

**Keywords:** COVID-19, MCC, SARS-CoV-2, microbiomes

## Abstract

Although the COVID-19 pandemic is caused by a single virus, the rest of the human microbiome appears to be involved in the disease and could influence vaccine responses while offering opportunities for microbiome-directed therapeutics. The newly formed Microbiome Centers Consortium (MCC) surveyed its membership and identified four ways to leverage the strengths and experience of microbiome centers in the response to the COVID-19 pandemic. To meet these needs, the MCC will provide a platform to coordinate clinical and environmental research, assist with practical obstacles, and help communicate the connections between the microbiome and COVID-19. We ask that microbiome researchers join us in these efforts to address the ongoing pandemic.

## Abstract (precis)

Although the COVID-19 pandemic is caused by a single virus, the rest of the human microbiome appears to be involved in the disease and could influence vaccine responses while offering opportunities for microbiome-directed therapeutics. The newly formed Microbiome Centers Consortium (MCC) surveyed its membership and identified four ways to leverage the strengths and experience of microbiome centers in the response to the COVID-19 pandemic.

## COMMENTARY

The COVID-19 pandemic is a once-in-a-century public health crisis, currently having infected 52 million people worldwide and resulting in more than 1.3 million deaths. Although caused by a single microbe (the SARS-CoV-2 virus), COVID-19 is likely influenced by the rest of the microbiome as evidenced by gastrointestinal manifestations, viral persistence in the gut, and risk factors associated with gut microbiome dysbiosis (1–3). Responding to this pandemic requires coordination. The Microbiome Centers Consortium (MCC) is a research network of academic microbiome centers, whose goal is to share strategies and accelerate knowledge transfer to promote the holistic study of microbiomes (4). The MCC hopes to leverage the strengths and experience of microbiome centers around the United States to help with the COVID-19 response, including coordinating availability of equipment, optimizing and testing protocols, advice on obtaining ethical (e.g., IRB) and regulatory (e.g., Emergency Use Authorization [EUA]) approval, and training personnel. Microbiome researchers also have the potential to help uncover the basic biology underlying SARS-CoV-2 host interactions and the development and treatment of COVID-19 disease. This is especially relevant in populations with higher levels of comorbidities for COVID-19 known to be linked to the microbiome, including diabetes, obesity, cardiovascular disease, and respiratory illnesses (1, 5, 6). The association between microbiome characteristics and therapeutic or vaccine responses is another area in which microbiome research could impact how we emerge from the pandemic. Our effort aims to coordinate and assist with practical obstacles as well, since many of our members are struggling with similar challenges (e.g., CLIA certification, IRB approval, and access to samples). In initial discussions among MCC participants and an online survey distributed to our members and their colleagues, we have identified at least four ways in which we can contribute.

**(i) Coordinate research efforts that focus on how the human microbiome might be involved in or affected by SARS-CoV-2 infection and/or COVID-19 disease (3, 7, 8)**. This might also include the development of microbiome-directed therapeutics to prevent or treat COVID-19 (9, 10). Many groups are currently limited by sample availability, due to difficulties in obtaining samples for microbiome analyses and fluctuations in infection numbers. Indeed, there is a strong willingness within our field to collaborate at this critical time. Resolving this bottleneck will greatly increase progress. Survey data from 26 MCC centers revealed that most (85%) survey respondents are involved in clinical studies and expressed interest in collaborating on shared samples. To start, the MCC is maintaining a web registry of ongoing SARS-CoV-2 projects that involve microbiome sampling (e.g., stool, saliva, and mucosa) and microbiota-directed interventions (e.g., probiotics and prebiotics) so that researchers can collaborate (microbiomecenters.org). Our members are also converging on similar obstacles and therefore can begin to make key recommendations for future studies. For instance, it is important to establish clinical studies so that human-subject approval documents and consent forms contain language that allows sharing specimens with other sites for future microbiome studies and a procedure for deidentification of subjects to reduce difficulties associated with confidentiality (e.g., HIPAA). The latter is particularly important in cases in which SARS-CoV-2 status or other Protected Health Information will be obtained. Another important consideration is translation of consent forms into appropriate languages for recruitment, especially in regions enriched for non-English-speaking populations. We call on microbiome researchers—even those not yet involved in SARS-CoV-2 research—to help coordinate research in their institutions to assess the importance of the SARS-CoV-2/microbiome nexus in various contexts.

**(ii) Coordinate efforts to help with regulatory lab extensions for clinical testing.** Many of our centers have extensive expertise in nucleic acid extraction and high-throughput methods that could be helpful to others. The regulations guiding this vary considerably by jurisdiction in the United States. In some locations, asymptomatic screening can be conducted purely on a research basis, although typically results cannot be returned to individuals, which decreases participation. In some states, research labs can apply to conduct clinical tests directly, in others they can operate under extension of the CLIA (Clinical Laboratory Improvement Amendments) license of an existing lab at the same address or even at a different address within the same institution, and in others they must apply for their own license. Complying with such rules is an arduous process, but not impossible, even for research labs with no prior clinical experience. The MCC will help to connect researchers with other groups that have already overcome these obstacles.

**(iii) Coordinate efforts for nonclinical research that tracks SARS-CoV-2.** Nonclinical SARS-CoV-2 research involves environmental samples from sewage and the built environment—samples for which many microbiome centers already have experience. SARS-CoV-2 monitoring in public spaces such as schools, universities, and military environments will continue to be important even after we deploy vaccines. Environmental studies typically do not fall under the auspices of human-subject/CLIA rules and may provide a far more feasible way for most microbiome centers to be involved in relevant research. Ongoing studies are evenly split between sampling sewage and the built environment, but surprisingly, fewer centers seem to be involved in these studies (54% of our survey respondents) than in clinical ones (85%), suggesting a large capacity for growth in this area. The growing SARS-CoV-2 wastewater-based epidemiology group has vibrant Slack and protocols.io hubs (https://www.covid19wbec.org/) enabling active resource sharing and method development. In addition, building on expertise in built environment microbiology (e.g., the Sloan Microbiology of the Built Environment network, which includes many MCC members) leverages a wealth of experience in low-biomass sampling, nucleic acid extraction, and careful controls. Studies of human-associated samples including cell phones, used masks, etc., fall into the same category and could be useful for defining procedures for disinfection. We encourage our community to apply their expertise to these types of studies and to share their protocols moving forward.

**(iv) Aid in communication with the public.** A goal of the MCC moving forward is to help sort through the “hope versus hype” of microbiome science for the public. Microbiome researchers can communicate the connections (or not) between the microbiome and COVID-19, and MCC members are committed to making themselves available to help translate this research. We also ask for help in gathering relevant studies for our continuously updated website, to direct researchers and the public to relevant studies and ongoing efforts.

In sum, we invite additional sites to join the MCC, and together as a community to help build the science base and workforce, to address not only this once-in-a-century pandemic but future ones. Connect with us on Slack (MCC-COVID on slack.com), on the web (microbiomecenters.org), and via email (microbiomecenters@gmail.com) to connect to other researchers, help answer questions, and share strategies and protocols.
